# Factors associated with urinary retention after vaginal delivery under intraspinal anesthesia: a path analysis model

**DOI:** 10.1007/s00192-023-05684-1

**Published:** 2023-11-24

**Authors:** Hong-yan Ren, Huan-fang Zhang, Yu-yi Chen, Tai-zhen Luo

**Affiliations:** 1 The Third Clinical College of Guangzhou Medical University, The Nursing College of Guangzhou Medical University, 195 West Dongfeng Road, Guangzhou, 510182 China; 2 Department of Obstetrics and Gynecology, Obstetrics, Guangdong-Hong Kong-Macao Greater Bay Area Higher Education Joint Laboratory of Maternal-Fetal Medicine, The Third Affiliated Hospital of Guangzhou Medical University, No. 63 Duobao Road, Guangzhou, 510145 China; 3 Department of Nursing, Guangdong Provincial Key Laboratory of Major Obstetric Diseases, Guangdong Provincial Clinical Research Center for Obstetrics and Gynecology, The Third Affiliated Hospital of Guangzhou Medical University, No. 63, Duobao Road, 510145 Guangzhou, China

**Keywords:** Influencing factors, Intraspinal anesthesia delivery, Painless delivery, Path analysis, Postpartum urinary retention

## Abstract

**Introduction and hypothesis:**

Women who have intraspinal anesthesia for delivery are more likely to experience postpartum urinary retention (PUR), which, if not recognized and treated promptly, can result in long-term urinary dysfunction. Many factors influencing PUR have been proposed, but no study has been conducted to investigate the relationship between them. This study is aimed at determining the influencing factors of PUR and to explore the relationship between them.

**Methods:**

A prospective, cross-sectional survey using self-made questionnaires was conducted among 372 puerperae in a Grade A hospital in Guangzhou, China, from April to September 2022. SPSS25.0 and AMOS24.0 were used for data analysis, and a path analysis model was established to determine the relationship between the influencing factors.

**Results:**

The incidence of PUR was 49.85%. Residence, the level of postpartum pain, and the change of postnatal urination position had a direct effect on PUR. Episiotomy and analgesic duration have both direct and indirect effects on PUR. Forceps delivery, perineal edema and oxytocin had an indirect effect on PUR. Variables could influence the occurrence of PUR by mediating the analgesic duration, episiotomy, postpartum pain level, and postnatal urination position changes.

**Conclusions:**

This study provides an empirical model to illustrate the relationship between PUR and related factors in women who delivered under intraspinal anesthesia. In future management, more attention should be paid to women who live in cities, have higher levels of postpartum pain, longer analgesic duration, higher grade of perineal edema, and received episiotomy, forceps delivery, and oxytocin during labor.

## Introduction

Postpartum urinary retention (PUR) is a common postpartum complication, which can be divided into two kinds, overt and covert. Overt PUR refers to the inability to urinate by oneself within 6 h of vaginal delivery, and covert postpartum urinary retention refers to spontaneous urination within 6 h postpartum, but the bladder residual urine volume after the first urination ≥150 ml [1]. Owing to differences in diagnostic criteria and study inclusion criteria, the reported incidence of postpartum urinary retention ranges from 0.2 to 45% [2].

Pathophysiological changes in the development of PUR have not been accurately concluded, but multiple factors related to the physiological, neurological, and mechanical processes occurring during pregnancy and vaginal delivery have been reported, and painless delivery has been noted as its independent influencing factor [2, 3], maybe related to the blockade of anesthetic drugs to relieve the pain, but also to inhibit the normal urination reflex, which affects the sensitivity of the bladder [4]. Painless delivery is widely used in Western countries but started late in China. In 2018, the National Health Commission of China issued the Notice on the Pilot Work of Labor Analgesia to promote the practice of labor analgesia. The latest data show that the labor analgesia rate in some pilot hospitals has reached more than 70% [5]. However, women delivered under intraspinal anesthesia have been reported to have longer first- and second-stage labor times, higher vaginal midwifery rates, and higher PUR rates [4]. In this study we intend to calculate the incidence of PUR and determine the influencing factors.

Previous analyses of factors affecting PUR have used univariate analysis and correlation analysis [2, 3]; interactions between the variables were not considered. The influencing factors of postpartum urinary retention are variable and interactive, path analysis can handle multiple independent variables and dependent variables, clear the total effect of each independent variable, direct effect, and indirect effect, and test the advantages of the fit of the whole model; therefore, in this study we planned to use path analysis to explore the relationship among factors influencing PUR.

## Materials and methods

### Study population

From April to September 2022, a convenient sampling method was adopted to select women who gave birth in a third-class A hospital in Guangzhou, Guangdong Province, China, and met the inclusion and exclusion criteria. The inclusion criteria were 28 to 42 weeks of gestation; voluntary intraspinal anesthesia delivery after physician evaluation and communication with the patient; and voluntary participation in the study. The exclusion criteria were prenatal patients with urinary disease and patients with mental disorders or who were unable to communicate. The elimination criteria were conversion to cesarean section during delivery; and wanting to withdraw from the investigator for any reason.

Path analysis requires calculation based on the sample size of no less than 150 cases and 10 to 20 occurrences of the observed variables [6]. Considering a 15% shedding rate, the sample size is about 357. In this study, 372 questionnaires were distributed, and 333 valid questionnaires were collected, with an effective recovery rate of 89.52%.

### Study variables

The general data questionnaire was designed to include nationality, educational background, marital status, residence, occupation and medical payment method. The questionnaire on the influencing factors of PUR after intraspinal anesthesia was obtained from the previous research results of the research group. It was designed based on the Delphi method and the theory of displeasure symptoms, the influencing factors of PUR included physiological, psychological, and environmental factors [7]. After two rounds of expert correspondence, the first level item was added: medical intervention factor; as this study was a single-center study, all women faced the same medical environment after delivery and ignored the first-level item environmental factor. The modified influencing factors questionnaire included a total of three first-level items and 31 second-level entries. First-level item physiological factors were Body Mass Index (BMI) before pregnancy, weight gain during pregnancy, age, number of births, prenatal activity, pregnancy complications, the second stage of labor time, total labor time, degrees of perineal laceration, neonatal birth weight, postpartum pain level, history of urinary retention, degrees of perineal edema, and degrees of hemorrhoid edema. First-level item medical intervention factors were prenatal bed urination training, prenatal pelvic floor muscle exercise, number of catheterizations during labor, free position delivery application, oxytocin, water sac labor induction, manual rotation of the fetal head, episiotomy, forceps delivery, vacuum extractor of the fetal head, analgesic duration, and use of antispasmodic drugs during childbirth. First-level item psychological factors were depression, anxiety, psychological support, the change of postnatal urination posture, and the mastery of PUR-related knowledge.

The relevant indicators are defined as follows:

Body Mass Index before pregnancy classification was: low weight, BMI <18.5; normal weight, 18.5 to 24; overweight, 24–28; and obesity, BMI ≥28 [8]. Weight gain during pregnancy was divided according to maternal BMI, 11.0 to 16.0 kg for low weight; 8.0 to 14.0 kg for normal weight; 7.0 to 11.0 kg for overweight; and 5.0 to 9.0 kg for obesity [8]. Maternal body weight gain in the range is recorded as normal body weight gain during pregnancy, less than the range of increase range as insufficient body weight gain during pregnancy, and more than the range of increase range as excessive body weight gain during pregnancy.

Degrees of perineal laceration [9] were degree I, perineal skin and (or) vaginal mucosa damage; degree II, with perineal muscle damage; degree III, injury involving the anal sphincter; degree IV, damage to the internal and external anal sphincter and involvement of the rectal mucosa.

Degrees of perineal edema [10] were no edema; degree I, mild perineal swelling, skin pattern; degree II, perineal swelling to shiny skin, skin pattern disappeared; and degree III, perineal swelling to clear skin, and swelling of the peripheral labia.

Degrees of hemorrhoid edema [11]: Degree I, no edema; degree II, mild, mild edema, can be incorporated into the anus; degree III, moderate, obvious edema, pain; degree IV, severe, obvious edema, pain, with erosion and necrosis of internal hemorrhoids.

The postpartum pain level was assessed by the simple pain numerical rating scale (NRS) with 0–10 points. Patients were required to choose the number best representing their pain intensity from 0 to 10 points, with 0 painless pain, 1–3 indicating mild pain, 4–6 moderate pain, 7–9 severe pain, and 10 excruciating pain.

Maternal depression and anxiety were assessed using the Hospital Anxiety Depression Scale (HADS), the scale established in [12] by Zigmond et al. Cronbach's α coefficients of the Chinese subscale for anxiety and depression were 0.762 and 0.787 respectively, showing good reliability. Between the two subscales, 0–7 was classified as no depression or anxiety, 8–10 was classified as suspected depression or anxiety symptoms, and 11–21 was classified as certain depression or anxiety symptoms [13].

The prenatal activity of women used the physical activity questionnaire during pregnancy (PPAQ) proposed by Chasan-Taber et al. in [14]. The Chinese version of the questionnaire included 31 items, the content validity was 0.940, and the retest reliability was 0.944 [15]. According to the Canadian guidelines for physical activity during pregnancy, moderate physical activity for at least 150 min per week was recommended as the standard of physical activity during pregnancy [16].

The mastery of PUR-related knowledge was evaluated by self-designed questions. When puerperae thought that they had PUR knowledge and knew the importance of early postpartum urination, they needed to answer three multiple choice questions, which covered the definition, influencing factors, and preventive measures of PUR. Puerperae who had PUR-related knowledge were recorded as having 0 points, those who did have PUR-related knowledge, but got 0 correct, were recorded as having 1 point, if they got 1 correct this was recorded as 2 points, and so on; when 3 questions were all correct, this was recorded as 4 points.

In this study, a portable, non-invasive transabdominal bladder ultrasound device (Bladderscan® PBSV4.1; Mianyang Meike, China) was used to scan the bladder of a woman immediately after her first postpartum urination. The residual urinary volume after the first spontaneous urination was ≥150 ml and was recorded as PUR.

### Ethical approval

This study was approved by the ethical review body of the Third Affiliated Hospital of Guangzhou Medical University. Before data collection, consent was obtained from the relevant departments, and all pregnant women participating in the survey signed written informed consent.

### Data collection

Considering the energy and cooperation of the puerperae, the questionnaire was divided into two parts. One part was filled in by the puerperae, and the data included were those not available from the medical data, such as postpartum pain level, psychological support, prenatal activity, etc. The other part was filled in by the investigator, and included data that can be obtained directly from the maternal medical data, such as whether there is an episiotomy, the duration of the second stage of labor, etc. Within 6 h of delivery, puerperae who voluntarily participated in were surveyed; the purpose, significance, and questionnaire filling method were explained; and the questionnaires were filled in independently. If needed the puerpera was assisted by the investigator and the questionnaires were recalled on the spot. If there were any omissions, they were completed immediately.

### Data and statistical analysis

Data processing was performed using SPSS25.0, and all continuous variables were converted into categorical variables before analysis. Using the frequency and composition ratio description, categorical data were compared using the Chi-squared test and nonparametric test, and *p*<0.05 was considered a statistically significant difference.The path analysis model was established and modified using AMOS24.0, and estimated using the maximum likelihood method, when the Chi-squared degree of freedom ratio (χ^2^/df) <2, comparing the fitting index (CFI) and the Tucker–Lewis index (TLI) >0.90, the root mean square error of approximation (RMSEA) <0.05, and was considered a good model fit [17].

## Results

### Descriptive and univariate analysis of data

This study included 333 maternal intraspinal anesthesia delivery, 166 (49.85%) had PUR. There were statistically significant differences in the incidence of PUR among different places of residence, degree of perineal laceration, newborn birth weight, postpartum pain level, degree of perineal edema, analgesic duration, presence or absence of oxytocin, episiotomy, forceps delivery, and changes in urination posture.

Specific maternal general data and possible factors affecting postpartum urinary retention are detailed in Table 1.

**Table 1:** General data and influencing factors of maternal anesthesia

			*n*	No PUR, *n*=167(50.15%)	PUR, *n*=166(49.85%)	x^2^/Z	*p*
1	Age	<35	276 (82.88)	136 (40.84)	140 (42.04)	0.494	0.482
		≥35	57 (17.12)	31 (9.31)	26 (7.81)		
2	Nationality	Han nationality	328 (98.50)	165 (49.55)	163 (48.95)	0.209	0.685
		Not Han nationality	5 (1.50)	2 (0.60)	3 (0.90)		
3	Educational background	Junior middle school	46 (13.81)	26 (7.81)	20 (6.01)	5.767	0.217
		High school/technical secondary school	27 (8.11)	13 (3.90)	14 (4.20)		
		Junior college	102 (30.63)	58 (17.42)	44 (13.21)		
		Regular college course	140 (42.04)	64 (19.22)	76 (22.82)		
		Graduate student or above	18 (5.41)	6 (1.80)	12 (3.60)		
4	Marital status	Unmarried	3 (0.90)	2 (0.60)	1 (0.30)	1.329	0.514
		Married	329 (98.80)	165 (49.55)	164 (49.25)		
		Divorced	1 (0.30)	0 (0.00)	1 (0.30)		
		Widowed	0 (0.00)	0 (0.00)	0 (0.00)		
5	Place of residence	Rural area	29 (8.71)	20 (6.01)	9 (2.70)	4.498	0.034
		City	304 (91.29)	147 (44.14)	157 (47.15)		
6	Occupation	National civil servant/professional and technical personnel	31 (9 .31)	17 (5.11)	14 (4.20)	1.964	0.854
		Worker	205 (61.56)	102 (30.63)	103 (30.93)		
		Self-employed/freelancer	39 (11.71)	21 (6.31)	18 (5.41)		
		Farmer	1 (0.30)	1 (0.30)	0 (0.00)		
		Unemployed	13 (3.90)	6 (1.80)	7 (2.10)		
		Other	44 (13.21)	20 (6.01)	24 (7.21)		
7	Mode of medical payment	Maternity insurance	279 (83.78)	140 (42.04)	139 (41.74)	2.341	0.310
		At one's own expense	46 (13.81)	25 (7.51)	21 (6.31)		
		At public expense	8 (2.40)	2 (0.60)	6 (1.80)		
8	Number of deliveries	0 times	223 (66.97)	105 (31.53)	118 (35.44)	3.684	0.298
		1 time	95 (28.53)	53 (15.92)	42 (12.61)		
		2 times	12 (3.60)	8 (2.40)	4 (1.20)		
		≥3 times	3 (0.90)	1 (0.30)	2 (0.60)		
9	Pre-pregnancy BMI	<18.5	72 (21.62)	41 (12.31)	31 (9.31)	6.062	0.109
		18.5 to 24	210 (63.06)	101 (30.33)	109 (32.73)		
		24–28	44 (13.21)	19 (5.71)	25 (7.51)		
		≥28	7 (2.10)	6 (1.80)	1 (0.30)		
10	Weight gain during pregnancy	Insufficient	31 (9.31)	16 (4.80)	15 (4.50)	0.043	0.979
		Normal	165 (49.55)	83 (24.92)	82 (24.62)		
		Too much	137 (41.14)	68 (20.42)	69 (20.72)		
11	Pregnancy activity	Not up to standard	307 (92.19)	154 (46.25)	153 (45.95)	0.000	0.987
		Reach the standard	26 (7.81)	13 (3.90)	13 (3.90)		
12	Pregnancy complications	No	135 (40.54)	66 (19.82)	69 (20.72)	0.144	0.704
		Yes	198 (59.46)	101 (30.33)	97 (29.13)		
13	Duration of second stage of labor	≤120 min	291 (87.39)	147 (44.14)	144 (43.24)	0.145	0.930
		≤180 min	34 (10.21)	16 (4.80)	18 (5.41)		
		>180 min	8 (2.40)	4 (1.20)	4 (1.20)		
14	Total duration of labor	≤720 min	230 (69.07)	121 (36.34)	109 (32.73)	1.798	0.180
		>720 min	103 (30.93)	46 (13.81)	57 (17.12)		
15	Degree of perineal tear	None	114 (34.23)	40 (12.01)	74 (22.22)	15.788	0.000
		Degree 1	215 (64.56)	125 (37.54)	90 (27.03)		
		Degree 2	4 (1.20)	2 (0.60)	2 (0.60)		
		Degree 3	0 (0.00)	0 (0.00)	0 (0.00)		
16	Birth weight of the newborn	<2,500 g	6 (1.80)	0 (0.00)	6 (1.80)	11.410	0.003
		2,500–4,000 g	322 (96.70)	167 (50.15)	155 (46.55)		
		≥4,000 g	5 (1.50)	0 (0.00)	5 (1.50)		
17	Level of postpartum pain	0 point, painless	0 (0.00)	0 (0.00)	0 (0.00)	28.144	0.000
		1–3 points, mild	128 (38.44)	70 (21.02)	58 (17.42)		
		4–6 points, moderate	156 (46.85)	89 (26.73)	67 (20.12)		
		7–9 points, severe	37 (11.11)	8 (2.40)	29 (8.71)		
		10 points, excruciating	12 (3.60)	0 (0.00)	12 (3.60)		
18	History of urinary retention	No	324 (97.30)	161 (48.35)	163 (48.95)	1.009	0.502
		Yes	9 (2.70)	6 (1.80)	3 (0.90)		
19	Degree of perineal edema	None	135 (40.54)	82 (24.62)	53 (15.92)	11.305	0.010
		Degree 1	73 (21.92)	33 (9.91)	40 (12.01)		
		Degree 2	37 (11.11)	13 (3.90)	24 (7.21)		
		Degree 3	88 (26.43)	39 (11.71)	49 (14.71)		
20	Degree of hemorrhoid edema	Degree 1	260 (78.08)	137 (41.14)	123 (36.94)	3.882	0.274
		Degree 2	56 (16.82)	24 (7.21)	32 (9.61)		
		Degree 3	16 (4.80)	6 (1.80)	10 (3.00)		
		Degree 4	1 (0.30)	0 (0.00)	1 (0.30)		
21	Antenatal bed urination training	No	289 (86.79)	149 (44.74)	140 (42.04)	1.732	0.188
		Yes	44 (13.21)	18 (5.41)	26 (7.81)		
22	Antenatal pelvic floor muscle exercise	No	245 (73.57)	125 (37.54)	120 (36.04)	0.310	0.857
		Once in a while	76 (22.82)	36 (10.81)	40 (12.01)		
		Often	12 (3.60)	6 (1.80)	6 (1.80)		
23	Number of catheterizations during labor	0 times	118 (35.44)	69 (20.72)	49 (14.71)	7.732	0.052
		1 time	162 (48.65)	79 (23.72)	83 (24.92)		
		2 times	42 (12.61)	15 (4.50)	27 (8.11)		
		≥ 3 times	11 (3.30)	4 (1.20)	7 (2.10)		
24	Free position labor	No	251 (75.38)	122 (36.64)	129 (38.74)	0.973	0.324
		Yes	82 (24.62)	45 (13.51)	37 (11.11)		
25	Oxytocin	No	151 (45.35)	85 (25.53)	66 (19.82)	4.168	0.041
		Yes	182 (54.65)	82 (24.62)	100 (30.03)		
26	Water balloon induction of labor	No	284 (85.29)	142 (42.64)	142 (42.64)	0.017	0.895
		Yes	49 (14.71)	25 (7.51)	24 (7.21)		
27	Manual turning of the fetal head	No	331 (99.40)	167 (50.15)	164 (49.25)	2.204	0.248
		Yes	2 (0.60)	0 (0.00)	2 (0.60)		
28	Episiotomy	No	234 (70.27)	134 (40.24)	100 (30.03)	15.937	0.000
		Yes	99 (29.73)	33 (9.91)	66 (19.82)		
29	Forceps delivery	No	323 (97.00)	167 (50.15)	156 (46.85)	10.372	0.001
		Yes	10 (3.00)	0 (0.00)	10 (3.00)		
30	Fetal head vacuum extractor	No	324 (97.30)	163 (48.95)	161 (48.35)	0.120	0.750
		Yes	9 (2.70)	4 (1.20)	5 (1.50)		
31	Duration of analgesia	<180 min	40 (12.01)	28 (8.41)	12 (3.60)	14.430	0.002
		180–360 min	110 (33.03)	59 (17.72)	51 (15.32)		
		360–600 min	112 (33.63)	56 (16.82)	56 (16.82)		
		≥600 min	71 (21.32)	24 (7.21)	47 (14.11)		
32	Use of antispasmodic medication during labor	No	332 (99.70)	166 (49.85)	166 (49.85)	0.997	1.000
		Yes	1 (0.30)	1 (0.30)	0 (0.00)		
33	Depression	≤7 points	280 (84.08)	143 (42.94)	137 (41.14)	0.707	0.702
		8–10 points	43 (12.91)	19 (5.71)	24 (7.21)		
		11–21 points	10 (3.00)	5 (1.50)	5 (1.50)		
34	Anxiety	≤7 points	284 (85.29)	148 (44.44)	136 (40.84)	4.169	0.124
		8~10 points	40 (12.01)	17 (5.11)	23 (6.91)		
		11–21 points	9 (2.70)	2 (0.60)	7 (2.10)		
35	Psychological support	No	180 (54.05)	97 (29.13)	83 (24.92)	2.190	0.139
		Yes	153 (45.95)	70 (21.02)	83 (24.92)		
36	Knowledge about PUR	No understanding	293 (87.99)	141 (42.34)	152 (45.65)	6.866	0.143
		Understand, but score 0 points	12 (3.60)	7 (2.10)	5 (1.50)		
		Understand, score 1 point	20 (6.00)	10 (3.00)	10 (3.00)		
		Understand, score 2 points	7 (2.10)	3 (0.90)	4 (1.20)		
		Understand, score 3 points	1 (0.30)	1 (0.30)	0 (0.00)		
37	Change in postnatal urinary position	No	290 (87.09)	157 (47.15)	133 (39.94)	14.286	0.000
		Yes	43 (12.91)	10 (3.00)	33 (9.91)		

### Correlation analysis

Before the path analysis, bivariate correlation was used to analyze the relationship between variables, postpartum pain level was directly related to perineal laceration degree, degrees of perineal edema, episiotomy, forceps delivery, and analgesic duration; analgesia duration was directly related to degrees of perineal laceration, oxytocin, and episiotomy; and episiotomy was directly related to perineal laceration; perineal edema was directly related to the change of postnatal urination posture. See Table 2 for details.

**Table 2:** Correlation analysis of the influencing factors

	Place of residence	Degree of perineal tear	Degree of perineal oedema	Change in postnatal urinary position	Oxytocin	Episiotomy	Forceps delivery	Birth weight of the newborn	Duration of analgesia	Level of postpartum pain
Place of residence	1	–	–	–	–	–	–	–	–	–
Degree of perineal tear	−0.034	1	–	–	–	–	–	–	–	–
Degree of perineal oedema	−0.002	−0.075	1	–	–	–	–	–	–	–
Change in postnatal urinary position	−0.040	0.058	0.138*	1	–	–	–	–	–	–
Oxytocin	0.082	−0.072	0.036	0.027	1	–	–	–	–	–
Episiotomy	0.014	−0.853**	0.106	−0.055	0.104	1	–	–	–	–
Forceps delivery	−0.008	−0.131*	0.024	0.037	0.019	0.194**	1	–	–	–
Birth weight of the newborn	−0.064	0.056	0.017	−0.043	−0.015	−0.062	0.003	1	–	–
Duration of analgesia	0.007	−0.220**	0.077	0.089	0.293**	0.266**	0.048	−0.024	1	–
Level of postpartum pain	0.030	−0.244**	0.125*	0.065	0.059	0.262**	0.296**	0.017	0.185**	1

*p<0.05; **p<0.01

### Path analysis

**Fig. 1:**
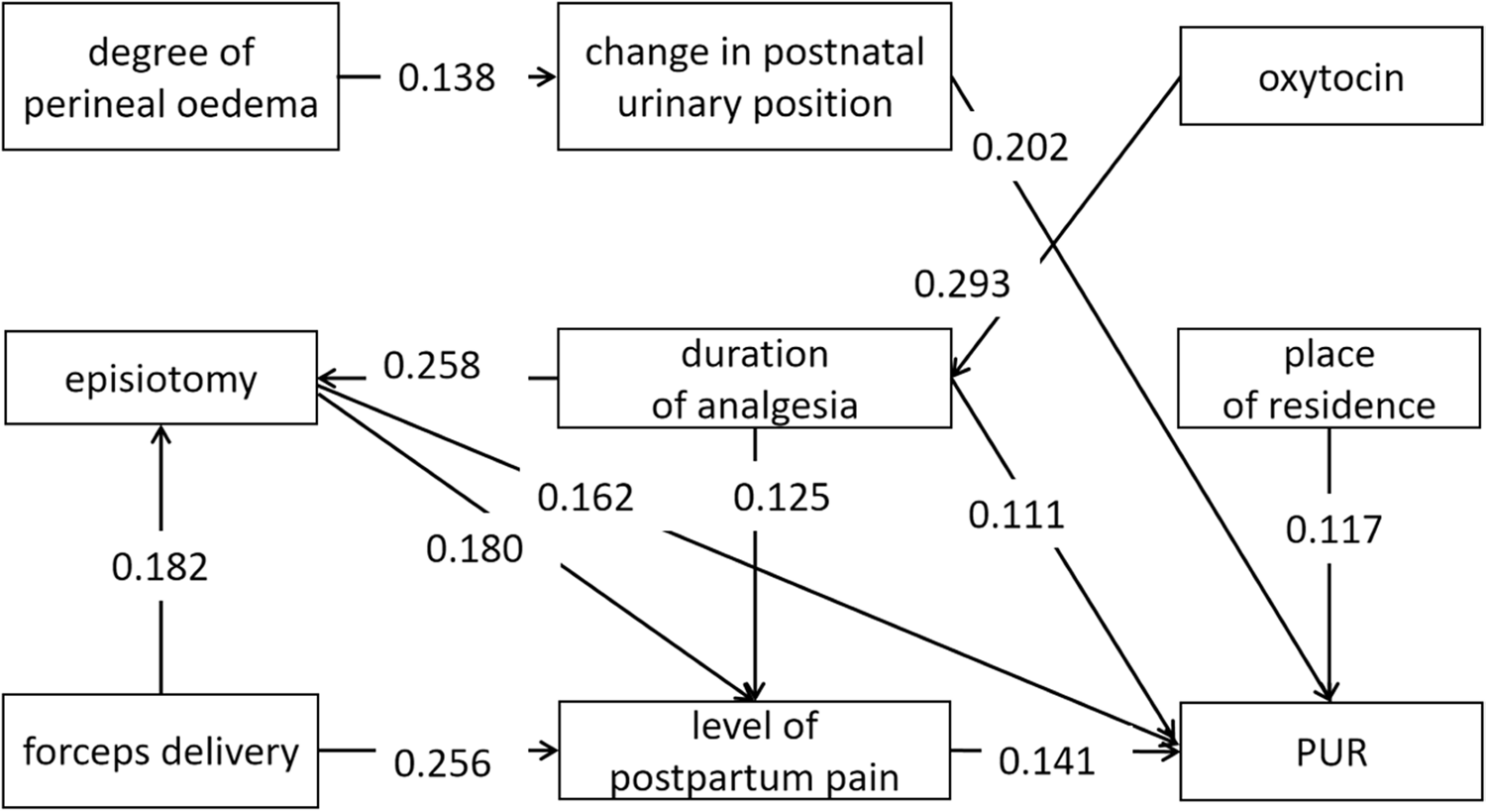
Path analysis model of postpartum urinary retention (*PUR*)-influencing factors in intraspinal anesthesia

Path analysis models were constructed based on correlation analysis, literature search, and clinical experience, and pathways lacking statistical significance and contrary to theory were removed to ensure parsimony and fit of the model. The final constructed model, Chi-squared=28.236 (*p*=0.250>0.05), CFI = 0.974, TLI = 0962, RMSEA = 0.023, 90% CI = 0.000–0.052), good model fit. Among them, postpartum pain level, change of postnatal urination posture, and maternal residence directly affect PUR; degree of perineal edema, forceps delivery, and oxytocin have an indirect impact on PUR; episiotomy and analgesic duration have both direct and indirect effects on PUR. The final path analysis model showed only statistically significant variables (Fig. 1, Table 3).

**Table 3:** Direct, indirect, and total effects of variables on the occurrence of postpartum urinary retention

	Direct effect	Indirect effect	Total effect
Degree of perineal oedema	–	0.028	0.028
Forceps delivery	–	0.070	0.070
Oxytocin	–	0.052	0.052
Place of residence	0.117	–	0.117
Episiotomy	0.162	0.025	0.187
Duration of analgesia	0.111	0.066	0.177
Level of postpartum pain	0.141	–	0.141
Change in postnatal urinary position	0.202	–	0.202

## Discussion

The incidence of PUR in women delivered under intraspinal anesthesia was 49.85%, which was higher than in previous reports, and the incidence of 8.1% to 47% in studies using the same diagnostic criteria was reported [1, 3, 18, 19]. Maybe because these studies used defferent criterias. But in this survey, all women accepted the intraspinal anesthesia delivery, anesthetic drugs act on the lumbar and sacrococcygeal nerve fibers, blocking the afferent and outgoing of bladder nerve impulse, thereby reducing bladder sensitivity and contractility, the coordination between bladder detrusor contraction and urethral sphincter relaxation was impaired [20].

Previous studies used multiple regression analysis to analyze the independent influencing factors of PUR. In women receiving instrumental midwifery, primiparity and prolonged second stage of labor were the independent influencing factors of the occurrence of PUR [21]. Episiotomy, epidural analgesia, and neonatal birth weight can affect the occurrence of covert PUR in vaginal delivery [3]. Prolonged second stage of labor, total labor over 700 min, instrumental midwifery, episiotomy, perineal laceration, and neonatal birth weight were independent influencing factors of urinary retention after vaginal delivery [18, 19]. In this study, we propose a model explaining the relationship between the influencing factors and the occurrence of PUR in women undergoing intraspinal anesthesia, and our study is also to our knowledge the first study to explore the relationship between PUR variables.

We found that in women who lived in the city, postpartum urination posture changed and higher postpartum pain levels were more likely to result in PUR. Studies on urban–rural differences in maternal complications found that rural women were relatively younger, and had a higher proportion of second children [22], nonprimiparous women may be more adapted to the effects of pregnancy and delivery on pelvic floor tissue, and younger women may have faster repair of pelvic floor tissue. The change of postpartum urination posture has the most prominent effect on PUR. In the urination position, squatting and sitting are considered to be relatively healthy urination positions, and there is no difference in urinary flow parameters and bladder residual urine volume after urination [23]. But considering the public health facility cleaning problem, 85% of women chose half squatting rather than sitting when using public squatting toilets, which led to a 21% decrease in mean urine flow and a 149% increase in residual urine volume after urination [24]. Also, as shown from the study by Rane and Iyer [25], compared with a supine position, squatting can passively increase the intra-abdominal pressure, so that the pelvic floor muscle resistance disappears, contributing to faster and smoother urination. Therefore, when the postpartum urination posture changes because of the toilet environment, with the wound pulling during squatting and postpartum hypoglycemia, the puerpera may be affected by the psychological inadaptation and the physiological relaxation of the pelvic floor muscles and urinary sphincter, increasing the risk of PUR. Higher postpartum pain levels may lead to urethral hyperactivity to increase the risk of PUR, and other studies have also shown that pain affects the incidence of PUR [2]. In addition, the postpartum pain level is also a mediating variable of the occurrence of PUR. Episiotomy, the use of forceps midwifery, and analgesic duration can all increase the risk of PUR by strengthening the postpartum pain level.

Episiotomy and analgesic duration, as mediating variables, had both direct and indirect effects on the occurrence of PUR. Episiotomy is a commonly used indicator in many studies [3, 26] that destroys the integrity of the perineal muscles and nerves, may directly affect the urination reflex, and may cause wound pain, subsequently affecting PUR, which is consistent with the results of this study. Most studies considered the effect of labor analgesia on PUR, and considered that labor analgesia is an independent influencing factor of PUR, but did not further explore the effect of analgesic duration. Considering that the longer analgesia occurs, the more narcotic accumulates in the body, the greater the inhibition of the voiding reflex. This was confirmed by a study on postoperative urinary retention, which showed an 11% increase for every 10-min extension of surgery and anesthesia [27]. The American College of Obstetricians and Gynecologists' 2019 guidelines for obstetric analgesia and anesthesia practice state that in the absence of contraindications to labor analgesia, the maternal requirement is the timing of labor analgesia [28]. In this study, labor analgesia began when the orifice of the uterine opening was 2–3 cm, and stops at 2 h postpartum, when the epidural catheter is removed; thus, analgesic duration can greatly affect delivery time. Considering the longer the analgesic time, the longer the delivery, the longer the pressure on the pelvic floor tissue, the greater the damage, which may mediate the postpartum pain level to affect PUR. In addition, analgesic duration can also influence PUR by mediating the use of episiotomy. For puerperae, longer analgesic duration, the cumulative effect of anesthetic drugs, and the long-term fetal pressure on the pelvic floor tissue may lead to decreased elasticity of the pelvic floor tissue and are not conducive to delivery. Therefore, in consideration of maternal and infant safety, extended analgesia and labor duration may increase the use of episiotomy and thus affect PUR.

The incidence of PUR was higher in women with a higher degree of perineal edema, forceps delivery, and oxytocin during labor. In the study by Cao et al. [26], perineal edema was an independent risk factor for PUR, and PUR was associated with mechanical obstruction of the urethral opening caused by perineal edema [27]. The results of this study show that perineal edema has an indirect positive effect on PUR; by mediating the change of postpartum urination posture, the effect value is 0.028. Perineal edema degree higher maternal may feel discomfort and worry about the wound during squatting due to the friction and squeezing of the edema skin and forced to use the unhealthy urination position and affect the bladder emptying.

Forceps delivery can indirectly affect the occurrence of PUR by mediating the use of episiotomy and the level of postpartum pain. Many studies on PUR have indicated that forceps delivery is an independent influencing factor of PUR, which is related to increased perineal and vaginal trauma. Women who receive instrument delivery have more damage caused by perineal strain and traction during delivery, and the resulting nerve anoxia or edema leads to reduced postpartum bladder emptying [29]. One study found that at least one of every four women with forceps had labor injuries [30]. Moreover, the forceps delivery has certain requirements for the use of space, and the simultaneous episiotomy has greater operation flexibility, which is conducive to the faster fetal delivery in emergency situations such as fetal distress. Therefore, the forceps delivery may increase the utilization rate of episiotomy. Birth injuries caused by forceps delivery or episiotomy caused by its mediation may affect the level of postpartum pain and thus cause reflex urethral spasm leading to urination disorders. Some studies have shown that oxytocin can reduce the total and the first stages of labor, and the effect of the second and third stages of labor is not significantly different [31]. This seems contrary to the results of this study, in which oxytocin had an indirect positive effect on PUR and could affect PUR by mediating the duration of analgesia. Analysis of the reason may be that in the study of oxytocin influence on delivery time, labor time is a continuous variable in the study, and in our study, the labor time is represented by analgesic duration, which is calculated as a categorical variable. Oxytocin is applied to uterine contraction fatigue and labor stagnation. Women who need to use oxytocin during labor have already had a prolonged labor, so they are more likely to be included in the group with longer analgesic duration, thus influencing the occurrence of PUR through mediating analgesic duration.

The advantage of this study is that it included a large number of homogeneous women receiving intraspinal anesthesia for delivery. All previous studies have considered labor analgesia as an independent influencing factor for PUR after vaginal delivery. This study explored the effect of analgesia duration on PUR and extended the influencing factor of PUR beyond obstetrics. It was found that place of residence and postnatal urination position also affected postnatal urination. In addition, the factors of PUR interact. Our study filled the gap in the structural relationship between variables and identified the direct and indirect relationships between them.

This study also has some limitations. In the Delphi expert correspondence, the impact of environmental factors on PUR was unanimously agreed by all experts, but this study was a single-center study and could not verify the effect of this variable.

## Conclusions

Almost half of women with intraspinal anesthesia delivery had urinary retention. The model was first established that illustrates the relationship between PUR and related factors in intraspinal anesthesia delivery. It is suggested that in the future management of PUR, more attention should be paid to the women who live in cities, have higher levels of postpartum pain, longer analgesic duration, higher degree of perineal edema, episiotomy, forceps delivery, and oxytocin during labor.
